# Distance Learning Support Measures for Teachers in Poland during the COVID-19 Pandemic

**DOI:** 10.3390/ijerph19138031

**Published:** 2022-06-30

**Authors:** Karina Cicha, Paulina Rutecka, Mariia Rizun, Artur Strzelecki

**Affiliations:** 1 Department of Communication Design and Analysis, University of Economics in Katowice, 40-287 Katowice, Poland; karina.cicha@ue.katowice.pl; 2 Department of Informatics, University of Economics in Katowice, 40-287 Katowice, Poland; paulina.rutecka@ue.katowice.pl (P.R.); artur.strzelecki@ue.katowice.pl (A.S.)

**Keywords:** higher education, universities, distance learning, hybrid education, academic teachers, COVID-19 regulations

## Abstract

The COVID-19 pandemic made higher education institutions switch to distance learning in a very short period of time. The situation was challenging not only for universities themselves but also for the students and teachers. Some universities did not have the means, in terms of infrastructure, for a smooth transition to distance learning. Some teachers were not prepared for the extensive usage of ICT in their work. The pandemic developed dynamically, and it made it extremely difficult for both governments and universities to plan and implement firm solutions on how to conduct the teaching process. The presented paper focuses on the situation of Polish higher education institutions between March 2020 and March 2022. It reviews legal acts and ordinances introduced in the stated period, which focused on the sustainability of the teaching process, countermeasures for the spread of COVID-19 and the implementation of distance learning. The case of the University of Economics in Katowice, Poland, is used to show the correlation between governmental legal acts and those introduced by the university as part of the COVID -19 spread prevention and teaching process support.

## 1. Introduction

Since the COVID-19 pandemic began in 2019 and was globally declared by the WHO in March 2020 [1], schools of all levels around the world have shifted to different teaching models: from remote learning to hybrid learning; from complete lockdown and suspension of teaching to short periods of time when schools are open and functioning as usual. The practice defined previously as emergency remote teaching—ERT [2], a type of distance learning [3]—soon became a common global practice [4]. It should be emphasized that remote teaching, introduced to schools rapidly as a countermeasure against the spread of SARS-CoV-2, had an impact not only on students [5] but also on the teachers [6].

According to a UNESCO report, on 1 April 2020, schools and higher education institutions (HEIs) were closed in 185 countries, which affected 1,542,412,000 learners [6]. The number reflects one fifth of the entire global population. The school situation is still being monitored by UNESCO, and the data about school closures are publicly available [7]. However, as reports and studies show, after two years of the global pandemic, there are still students deeply affected by its consequences [8]. The influence of the COVID-19 pandemic on education relates not only to ways of conducting the teaching process, e.g., usage of ICT [9,10], workload, materials distribution among students [11], or interactions between teachers and pupils [12], but also to the welfare of all parties involved in the process, e.g., psychological pressure or sense of isolation [2].

The adaptation to the new educational reality at the beginning of the pandemic was a challenge. What seemed to be a temporary solution has lasted for two years. This can be easily observed in higher education institutions, where, for instance, master’s degrees last two years; as such, there are now students who started and graduated their study programs entirely remotely, which is a visible sign of the digital transformation of education [13].

The digitalization of the education process sped up after the COVID-19 pandemic began. Higher education institutions worldwide use digital technology in the teaching process [14]. Face-to-face contact between teachers and students has been replaced by computer-mediated communication and digital interactions [15]. Exams are conducted online through such solutions as Google Forms or other adequate software [16]. The situation of HEIs, however, is still dynamic due to an unsure development of the pandemic itself, as well as potential governmental decisions.

Of particular interest when discussing the changes introduced to the teaching process are the digital competencies required of teachers and professors [17] for the education process to not be suspended. When defining digital competencies, it is necessary to not only emphasize the ability to use computer technology to communicate, acquire information, or critically analyze media messages. The term “digital competencies” includes digital literacy [18,19] as well as media literacy [20]. Therefore, to be digitally competent is to obtain skills allowing one to be fluent in fields such as technology, language, processes of interaction mediated by computers, production of media messages, the aesthetics of audiovisual messages, and the critical analysis of the media content [9,21]. For teachers, it is crucial not only to possess those skills but also to implement them and use them in the practice of teaching.

Since the COVID-19 pandemic started, Polish educational institutions have struggled to sustain the teaching process in all school types in the country. It was equally important to ensure access to education as well as to prevent the spread of the virus and follow governmental instructions. Those factors influenced the shape of the educational process in Poland in the last two years. Higher education institutions are a peculiar case. On the one hand, they need to follow governmental guidelines; on the other, a certain level of autonomy is allowed, so certain decisions about the organization of the teaching process are up to university authorities. On the national level, certain types of HEIs, such as public economics and business schools in Poland, are united in a consortium, and their rectors make decisions that apply to only those particular types of schools. The COVID-19 pandemic was a trial for universities, authorities, and students. However, what was most important was that all decisions about the organization of the teaching process undertaken over the last two years needed to be applied following nationwide governmental guidelines.

The novelty of this research relates to its thematic scope. Firstly, there are no publications analyzing the institutional process of implementing distance learning at universities in Poland. Secondly, the example of the University of Economics in Katowice, Poland, can be considered close to a representative one because most universities introduced similar measures, due to the fact that they were consulted in the National Conference of Rectors of Academic Schools in Poland (Polish: KRASP), as well as in other associations of rectors. Finally, the conducted study is a starting point for comparing the activities implemented at universities in Poland with those undertaken at universities in other countries.

Therefore, the main objective of the presented paper is to analyze the situation of Polish higher education institutions based on the example of the University of Economics in Katowice in relation to significant legal acts on three levels: the national level, the economic universities consortium level, and the single university level. To achieve the set objective, the authors raise five research questions (RQs) that are answered in the paper:

RQ1: What regulations were issued by the Ministry of Education and Science of Poland related to distance learning during the COVID-19 pandemic?

RQ2: What regulations were issued by the Conference of Rectors of Academic Schools of Poland related to distance learning during the COVID-19 pandemic?

RQ3: What actions were taken by higher education institutions in Poland during the COVID-19 pandemic?

RQ4: What stages of introducing distance learning during the COVID-19 pandemic in Poland can be distinguished?

RQ5: What kind of support did higher education institutions in Poland provide to their teachers in the process of shifting to distance learning during the COVID-19 pandemic?

The paper is divided into five sections. Section 2 presents methods used to find and categorize the legal acts as well as guidelines introduced since 2020 that have had an impact on the functioning of Polish universities. Section 3 presents changes in the functioning of HEIs in accordance with particular legal acts introduced to sustain the teaching process. Section 4 focuses on discussing the changes introduced into the teaching process in other countries. Section 5 summarizes the conclusions about the analyzed topic.

## 2. Materials and Methods

The method chosen for this study is a form of simplified content analysis, but without the keyword coding process. The purpose of such analysis is to identify the most important recommendations and classify actions taken by the European Union into stages corresponding to the development of the pandemic situation.

The review of the situation at Polish higher education institutions, which the authors conducted in this paper, can be divided into six major steps. In step 1, the authors searched the database of legal acts related to distance education during the COVID pandemic using the official website of the Journal of Laws of the Republic of Poland at [22], where all adopted normative acts are published in electronic form. Using the filtration module on the above page, the authors selected the legal acts published by the Minister of Science and Higher Education and the Minister of Education and Science in the period from 20 March 2020, to 20 March 2022.

In step 2, the authors also reviewed publications on the websites of the Ministry of Science and Higher Education and the Ministry of Education and Science. Apart from the legal acts mentioned above, the Ministry of Science and Higher Education issued 230 information notes and guidelines (from 13 March 2020, to 30 December 2020), with 115 of them containing “covid” as a keyword. The Ministry of Education and Science issued 915 information notes (from 1 January 2021, to 20 March 2022), with the “covid” keyword contained in 450 of them. All the articles (565) with “covid” were thoroughly reviewed by the authors to find guidelines for remote education and the overall functioning of higher education institutions. Findings of steps 1 and 2 are presented in the Section 3.1.

In step 3, the websites of the Conference of Rectors of Academic Schools in Poland and the Conference of Rectors of Economic Universities were reviewed for publications from 13 March 2020, to 20 March 2022, with the “covid” or “coronavirus” topics. The review results are presented in the Section 3.2.

The Section 3.3 contains the results of steps 4–6 of the review procedure. In step 4, the ordinances of the rector of the University of Economics in Katowice were reviewed [23]. As of 20 March 2022, 15 ordinances and 15 announcements from the rector were issued.

Step 5 includes a search through the website of the University of Economics in Katowice (publications from 13 March 2020, to 20 March 2022). In the selected period, 468 news articles were issued in the “University/News” section. The titles of all the publications were analyzed, and those related to distance learning during the COVID-19 pandemic were read thoroughly.

Finally, in step 6, the authors went through the content of all “UE Katowice Newsletter” emails obtained from 13 March 2020, to 20 March 2022. In total, 56 “Newsletter” emails concerning COVID-19 were sent by the University of Economics in Katowice to its employees.

Table 1 presents the number of messages according to the type issued by each authority, along with the number of communications related to teaching and to COVID-19.

## 3. Findings

### 3.1. National Ordinances

The state of the COVID-19 epidemic in Poland was announced on 20 March 2020 [24]. Until 1 January 2021, the following functions operated separately in Poland: the Ministry of National Education (responsible for primary and secondary schools) and the Ministry of Science and Higher Education (responsible for HEIs). From 1 January 2021, these ministries were merged into one: the Ministry of Education and Science. The search of legal acts published by the two Ministries within the indicated period (March 2020–March 2022, as stated above) showed that 187 legal acts were published, including 147 regulations, of which 57 were related to counteracting COVID-19 through changes in education. A time-lined summary of the regulations is presented below in Table 2. 

The first regulation on the suspension of teaching activities conducted by universities throughout the country (Journal of Laws of 2020, item 405) was published on 11 March 2020 [25], even before the announcement of the pandemic in Poland. This regulation suspended full-time education at all levels at universities (bachelor’s, master’s, doctoral, courses and training organized within the university, postgraduate studies, and others) and introduced the possibility of conducting classes during the suspension period via the use of distance learning methods and techniques.

The ordinance of 23 March [26] suspended full-time education for the next two weeks. In this regulation, the Minister stated that institutions that have appropriate infrastructure may undertake distance learning; however, it was up to university authorities to decide.

Subsequent regulations concerned the suspension or reinstatement of stationary education in subsequent periods. Often the regulation contained information on exceptions; for example, classes that could not be conducted online could be conducted periodically at the universities. Furthermore, certain classes for certain groups of students in selected semesters could be conducted at the university. In each of these regulations, the Minister stated that decisions on the form of education were to be made by the rector of the university and that the regulations only specify when it is categorically impossible to conduct full-time education.

More detailed information and guidelines were published on the website of the Ministry of Science and Higher Education [27] and then on the website of the Ministry of Education and Science [28]. On 13 March, the Ministry’s website published “Recommendations of the Ministry of Science and Higher Education” [29], in which the Ministry recommended that meetings with students should be organized using online communication platforms in the form of video conferences or webinars. At that time, it was also recommended that universities join the process of creating and sharing didactic materials in digital form under CC licenses. The Ministry also encouraged the use of open educational resources for universities created by other universities.

On 26 March 2020, through the website, the Ministry also informed about the possibility for universities to conduct exams using information technology [30]. On 27 March, the Ministry announced that, due to the introduction of remote education by most universities, recommendations were prepared regarding education provided with the use of distance learning methods and techniques [29,31]. The document contained recommendations regarding technological infrastructure, rules for the preparation and sharing of teaching materials, the rights and obligations of academic teachers and students, accounting for the teaching load of teachers, recognition of educational outcomes in the distance learning mode, an assessment of distance learning progress, and an assessment summarizing the effects of distance learning and diplomas. Within each of the above thematic sections, there were very general guidelines that provided autonomy to both university authorities and teachers. Among the recommendations, it is worth distinguishing those concerning universities and teachers.

According to the recommendations, universities should provide [31]:

- Technological infrastructure;
- Remote learning tools in accordance with applicable laws;
- Teachers support in the preparation of teaching materials;
- Supervision over the prepared teaching materials.

At that same time, the recommendations obligated teachers to:

- Prepare and provide students with educational materials in digital form;
- Develop and provide students with a description of the assumed educational outcomes and methods of their verification;
- Provide students with the exact teaching plan;
- Prepare teaching materials ensuring each student’s workload during classes is consistent with the number of hours assigned in the plan and program of study;
- Regularly monitor and document the students’ learning processes.

On 1 April 2020, information was published on the Ministry’s website stating that a free course for educators was available to help train them in the field of online teaching and to conduct remote lessons on the educational platform navoica.pl [32]. Enrollment for the course was possible until 6 May 2020, and the course was available until 1 July 2020. Currently, participation in the course is no longer possible.

The next recommendations of the Ministry appeared in September 2020 with respect to the start of a new semester at universities. These recommendations stated that the recommended method of educational organization was a mixed (hybrid) model. Furthermore, the recommendations also stated the guidelines of the sanitary regime during classes held on the university premises, the organization of apprenticeships, the functioning of libraries, and student dormitories. In later communications, there were no additional recommendations for universities in the context of conducting online classes or educational proposals for academic teachers.

### 3.2. Economic Universities Consortium Ordinances

Bearing in mind the need to develop a possibly homogeneous system of online classes during the COVID-19 pandemic, the rectors of Polish universities worked on a common position on this matter within rector associations. Rector associations, also called rector conferences, are voluntary associations in Polish universities [33] created to work out a common position for university authorities on important issues related to higher education in Poland. The University of Economics in Katowice is a member of, among others, the National Conference of Rectors of Academic Schools in Poland (Polish: KRASP), an association of almost 120 universities [34]. The university also operates within the Conference of Rectors of Economic Universities (Polish: KRUE) [35].

In regard to education during the COVID-19 pandemic, the KRASP published the following documents:

- “Preparation of the university for the new academic year 2020/2021 in relation to the COVID-19 pandemic in connection with the end of the period of limitation of the university’s functioning”;
- “Functioning of universities during the epidemic period in connection with SARS-CoV-2 virus infections, considering the re-limitation of the functioning of universities from 19 October 2020—summary of changes in legislation”;
- “Guidelines for the safe functioning of universities and other entities of the higher education and science system during the epidemic”.

There were no specific proposals in any of the above documents regarding the methods of conducting distance education or the preparation of the staff to conduct such classes. There was only one document regarding education in the COVID-19 period published by the KRUE: “On the organization of education in the summer semester in the 2020/2021 academic year”. The rectors agreed that education during the summer semester should be conducted using distance learning methods and techniques. However, the decisions regarding the particular methods and techniques were autonomous, and it was up to each rector to decide how to conduct education at the university under their management [36].

### 3.3. The University of Economics in Katowice Ordinances

The following section focuses on the example of the University of Economics in Katowice, the oldest business school in the Silesian Voivodeship. Due to having the highest population density in Poland, Silesia is one of the regions with the highest number of confirmed cases of COVID-19 [37]. Therefore, immediate action toward preventing the spread of COVID-19 needed to be undertaken. The reaction of the university authorities can be treated as a special form of communication in which the authorities directly or indirectly addressed the ordinances to the rest of academic community. As a form of indirect communication, the authors considered all the rector’s ordinances published between March 2020 and March 2022. As a form of direct communication between the authorities and the academic community, the authors considered all correspondence via email sent directly to teachers, administrative workers, and students at their university email addresses. Between 13 March 2020, and 14 April 2022, 52 emails were sent to employees and students of the University of Economics in Katowice as part of a newsletter, including information about conducting classes due to the COVID-19 pandemic, as well as countermeasures taken by the authorities to prevent the spread of the virus. Distance learning guidelines communicated directly and indirectly by EU authorities to teachers and students, and their implications for e-learning practices in the EU, are presented below in Table 3 in a timeline continuum, with the process divided into six stages of change.

In response to the regulation on the suspension of educational activities due to COVID-19 issued by the Ministry of Science and Higher Education on 11 March 2020, the rector of the University of Economics in Katowice issued an ordinance [23] suspending the educational process until March 25. In the same ordinance, an obligatory application of IT tools for the online education process was implemented. Moreover, the Crisis Management Committee [38] was set up to coordinate all activities related to the preventing the spread of COVID-19. According to the ordinance mentioned, one of the countermeasures against the spread of COVID-19 was the prohibition of gatherings at the university campus in groups of more than ten people. The suspension of the learning process was also prolonged until 10 April 2020. The changes introduced through this ordinance also affected the process of conducting bachelor’s and master’s exams that did not take place until 10 April. By the rector’s ordinance, issued to the public on 17 April, the exams at the end of the semester, including bachelor’s and master’s exams, were to be conducted remotely through online platforms. All the traditional forms of education were suspended at the University of Economics in Katowice until the end of the semester.

Even though the university authorities reacted very quickly, it soon turned out that the countermeasures against the spread of COVID-19 needed to be upheld and that all the actions that had already been undertaken required to be transformed into a firm strategy against the crisis. The transition to distance learning became a fact. Considering that the COVID-19 pandemic did not end in 2020, and looking back at the actions taken by the university, the process of transition to distant learning can be divided into six meaningful steps [38].

The first step can be dated back to around 13 March 2020, when all students, as well as teachers, were given access to online communication platforms, Moodle and Google Classroom, to sustain the learning process. It should be emphasized that both of these tools were already being used in the teaching process, but before March 2020, they were not obligatory. On the same day, the first email message to the academic community containing information about the suspension of classes and the start of conducting classes and consultations using ICT was sent. The email also provided a link to instructions according to which distance learning should implemented. First, all employees were ordered to set up an email account at the university’s domain, valid for Google tools. It was announced that education would be carried out using the following tools: Google for Education, Google Classroom, Moodle tools, and Google Drive. It was recommended that the Google package (Drive, Meet, Classroom) be used; however, the lecturers were free to use other tools such as Teams, Facebook, WhatsApp, and others, provided that their use for educational purposes was free or the university had an appropriate license. In addition, groups of email addresses, the so-called dean’s groups, were created, gathering all the students from a particular course. Thanks to this solution, the teacher did not have to invite each student separately but could send an invitation to the whole group. In the following message, from 21 March 2020, a link was sent that included a summary of the rules for the implementation of didactic classes [39] and instructional videos on the use of Google services [40].

The second step of the transition, dated back to around 30 March 2020, expanded the use of ICT to ensure live contact between teachers and students. Platforms such as Microsoft Teams, Google Meet, Google Hangouts, or Skype were broadly used by the teachers not only to conduct classes but also to contact students. Extended instructions were published on the university website to inform teachers and students how to use G Suite tools [38]. On 30 March 2020, the Vice-Rector for Education and Internationalization announced the university’s operation strategy with four stages of transition to distance learning [41]. On the same day, the introduction of the second stage of the transition to the distance learning process began, according to which teachers were to introduce at least one of the indicated forms of contact with students into their classes from 30 March:

- Connecting with a group of students/doctoral students during class hours via Google Meet or Chat, or alternative platforms for a short online meeting lasting 15–20 min, the purpose of which was to introduce students to the currently discussed issues;
- Sharing videos with information about individual classes and emphasizing the hours at which teachers would be available live in the form of online consultations;
- Using other forms of interactive communication with students.

Students were reminded that, under the regulations of studies, they were obliged to participate in classes, laboratories, language courses, and diploma seminars, which, in the case of online classes, meant active participation in remote forms of conducted classes.

The third step undertaken by the university authorities focused not just on conducting the classes or enabling students to contact their teachers, but mostly on the issue of preparing and conducting exams supported by online tools. According to the rector’s ordinance already mentioned, distance learning had been prolonged by the end of the summer semester of 2020. That meant that exams needed to be conducted remotely. The preparation started around 2 April 2020. On 3 April 2020, the message via newsletter was sent, which informed recipients about the preparation of detailed rules for conducting semester exams with the use of online solutions [38]. In addition, it was announced that computer rooms were available for teachers to enable them to conduct interactive online classes in a situation where they did not have the conditions to conduct classes from home. In the same email, students were sent a link to the opinion poll on e-learning in all degree programs organized by the university’s student parliament.

In the fourth step, bachelor’s and master’s exams were added to the general pool of exams that were about to be conducted. The first four diploma exams were conducted at UEK via Google Meet on 5 May 2020 [42,43]. According to the rector’s ordinance from 22 May 2020, distance learning was prolonged by the end of the semester. On 23 April 2020, the faculty was informed about the introduction of appropriate legal regulations necessary to ensure remote education and the verification of the learning outcomes [44]. Students were informed about the possibility of using the following platform: https://eu-citizen.science/ (accessed on 15 March 2022). The website “UE Katowice in the era of coronavirus” was launched as a form of aggregation of information on the functioning of universities during the pandemic.

Before the start of the winter semester, on 18 September 2020, the rules for the functioning of the university in the winter semester of the 2020/2021 academic year were announced [45]. According to these principles, distance education was to be maintained in full-time and part-time first- and second-degree studies, including physical education classes and education in postgraduate studies, courses, and training. However, hybrid teaching for first- and second-degree studies conducted in English was introduced. Education as part of international exchanges and doctoral studies was to take place in the university buildings. Unfortunately, a worsening of the pandemic caused all stationary activities to be suspended again on 15 October 2020 [46].

The fifth step of the transition was the assumptions introduced by the rector’s ordinance of 18 September 2020 [45]. According to that, teachers were to use the G Suite Platform regardless of whether their classes were conducted inhouse or remotely. The requirement for interactive contact with the student using Google Meet was also introduced. In the earlier stages, it was suggested but not compulsory. Previously, teachers could provide students with materials or recorded meetings in the form of videos. Platforms other than G Suite, such as Moodle or Microsoft Teams, could be used in support; however, links to all materials needed to be included in G Suite. By this ordinance, the G Suite platform was approved as the only officially valid online educational platform at the University of Economics in Katowice. Moreover, the teachers were obliged to invite a coordinator on the G Suite platform, who was to verify the quality of online classes. The inauguration of the 2020/2021 academic year, for the first time in the history of the university, took place entirely remotely. The 2020/2021 winter semester, except for a short period from 1 October to 15 October, was carried out entirely remotely.

An ordinance on the principles of the university’s operation in the summer semester of the 2020/2021 academic year was introduced on 8 February 2021 [47]. According to its content, remote education continued, with the proviso that the university may restore full-time teaching or introduce hybrid teaching. This possibility arose with the launch of a vaccination campaign among teachers and students. In the summer semester, classes were also conducted entirely online. An examination session was also held online. Exams could take place via Google’s G Suite, MS Teams, or other platforms. The use of the Safe Exam Browser (SEB) was recommended for the organization of exams using Moodle. SEB is a web browser environment developed to carry out e-assessments safely. The software turns any computer temporarily into a secure workstation. It controls access to resources like system functions, other websites and applications, and prevents unauthorized resources from being used during an exam [48]. Unfortunately, the Safe Exam Browser does not function properly with Windows 7, which caused problems with some students participating in the exams. In March 2021, training courses for academic teachers in the field of remote work security were organized.

The sixth step was the introduction of hybrid education in the winter semester 2021/2022. In accordance with the assumption of hybrid education at the University of Economics in Katowice, the stationary form may include exercises/laboratories, physical education classes, and English-language lectures in selected fields of study. Lectures, seminars, language courses, and consultations are to be held remotely. The division into types of classes is related to the number of students taking part in them. Lectures are conducted in larger groups of students and also in different study groups. Exercises and laboratories, on the other hand, are conducted for established study groups, which do not change throughout the study cycle. All English-language courses, and those at the doctoral school, are to be conducted in a full-time form. The usage of Google tools to support the teaching process remains the same. However, the possibilities of the Google educational package have changed. The package was renamed from G Suite to Google Workspace for Education. Google has introduced additional options for its platform’s use in education, such as virtual rooms in Google Meet and reports on student attendance and involvement, among others. Google conducted training on the new possibilities of the Google Workspace for Education package, the video record of which is available at events.withgoogle.com (accessed on 15 March 2022) [49]. In mid-December 2021, distance learning was reintroduced and lasted until 20 March 2022. The hybrid form of education was reintroduced on 21 March 2022, and, in accordance with the current regulation, is to last until the end of the semester.

In exceptional situations, such as sending a whole group of students or a teacher to quarantine due to contact with a person infected with COVID-19, the group could participate in remote classes, even if, as a rule, such classes were conducted at the university. The quarantine of teachers, however, turned out to be a bigger problem, as in these cases, the authorities usually did not consent to conducting classes online which were meant to take place in a classroom. This was due to the organization of the schedule for students, which, in accordance with the rector’s ordinance, assumed that, on certain days, classes for students would be conducted only online or only in class to adjust the system to a hybrid model of education.

At the time of writing (April 2022), no information from the University of Economics in Katowice has yet been disclosed to the public regarding the organization of the next academic year (2022/2023) and, thus, the form in which classes will be conducted. Furthermore, at that time, the Polish Ministry of Education and Science had not yet published any recommendations for the next semester.

In addition to the steps discussed above, it would be reasonable to mention that the education process at the University of Economics in Katowice was not the only one to be transformed into an online format. At the beginning of 2021, one of the degree programs at the Faculty of Informatics and Communication was evaluated by the Polish Accreditation Commission (Polish: PKA). A PKA visit to the university was planned for 2020. Due to the coronavirus outbreak, it was postponed. Later, at the beginning of 2021, it was decided to conduct the accreditation process online. Since no specific instructions for online accreditation had been issued before, both PKA members and employees of the University of Economics in Katowice engaged in the accreditation had to come up with solutions right away. A Google Drive folder was created to share materials between the university workers that prepared all the necessary documentation; then, another folder was created to provide PKA members with documentation; experts from the PKA attended a few online classes—they were provided with links to Google Meet in advance. Teachers, students, and administrative staff were also interviewed by the PKA online. Eventually, the accreditation was successful for this degree program, and, in addition to that, all the participants gained valuable experience in running the whole process online.

## 4. Discussion

In this work, we found that, since the beginning of the COVID-19 pandemic in March 2020 in Poland, the government, university consortia, and universities have slowly begun adapting to the new situation. It is clearly visible from the presented findings that the process of shifting from traditional forms of education to distance teaching was not smooth or easy. It took plenty of changes, acts, and ordinances in a timeframe of around two years to organize, prepare, and execute teaching in a distance or hybrid form.

At the beginning of the COVID-19 pandemic, the Polish government decided to suspend the teaching process and to shift to a distance learning method. This decision was similar to many other countries at that time. In addition, studies conducted after the initial phase of the COVID-19 pandemic show that institutions and teachers had to respond quickly to an unexpected transition from face-to-face to distance teaching [50]. 

The shift to distance learning, introduced by the government at an early stage of the COVID-19 pandemic countermeasures, was a challenge for teachers because it required applying different teaching methods. The literature focusing on the teacher struggles during the COVID-19 pandemic points out that teachers would like to revise their teaching for online delivery, and they hope that their efforts will result in good online teaching [51]. 

Distance education also requires introducing unified methods of conducting lectures and using the same, or at least similar, tools among university teachers. The Polish government, in one of the earliest regulations, recommended the Google for Education tools. The researchers focusing on ICT usage during the pandemic to indicate several areas related to the introduction of ICT into the teaching process. First, it was correlated with the teacher experiences in distance learning. However, in this regard, teachers’ prior experiences with the use of ICT seem to have played only a small role [52]. Second, studies show that, in this time of necessity, teachers showed adaptability in moving to online spaces [53]. Third, what is also emphasized in research, apart from the necessary minimum standards in order to ensure the continuity of the teaching process, a range of initiatives was developed or accelerated in response to the pandemic to reach individuals from disadvantaged backgrounds, including those with low skill levels or qualifications [54].

As the COVID-19 pandemic developed, the need for live contact between teachers and students was seen and introduced to the regulations (see Table 3, stage 2). Although teachers tended to adopt teaching strategies that reproduced standard classroom dynamics, the possibility of operating in this comfort zone generated a positive feeling about using technologies and a perception of increased digital skill mastery [55]. This is in line with our results, where observed changes also focused on reproducing standard classroom dynamics.

The beginning of the new school year in 2020 resulted in the need to improve the quality of distance learning. In order to do that, the Polish government introduced other regulations focusing on ICT used in the teaching process, as well as the interactivity of the learning process and the usage of hybrid teaching techniques (see Table 3, stage 5). This step was consistent with studies showing that information and communication technology (ICT) tools, particularly teacher digital competence and teacher education opportunities to obtain digital competence, were instrumental in adapting to online teaching during COVID-19 school closures [56]. However, there were also studies emphasizing that the greatest difficulties reported by educators were shortcomings in their training in digital skills, which made them perceive themselves as a having higher workload during the lockdown, along with negative emotions [57]; that digital literacy was not a reality that favors the teaching–learning process; and that a training program is urgently required for teachers to reach optimal levels of digital skill [58]. 

After all the regulations discussed in this paper were introduced, teachers grew to be better prepared to teach and examine in a mixed teaching environment. The teaching and learning process was supported with distance communication mixed with personal presence, adaptation, and compliance to the quickly changing regulations. The introduced regulations were effective. This work was compared with several situations in other European countries. We compared only the effects of the teaching process reported in other works since our study focused on support measures for teaching in distance learning.

An analysis of the information issued by the Ministry of Science and Higher Education and the Ministry of Education and Science of Poland, as well as by Polish higher education institutions (including the University of Economics in Katowice), supports the statement that the proper organization of educational processes during the COVID-19 pandemic were not neglected. Both the state and universities did their best to set the framework of distance education and to make sure teachers and students understood how the process was run. However, it turns out that, in this pandemic situation, much more attention was paid to students. Many research papers were published to analyze student opinions; universities conducted surveys to find out how students felt about e-learning, and academic teachers were constantly reminded of the guidelines they needed to follow in order to properly organize the educational process (e.g., how to deliver tasks to students, how to present oneself in front of students online, how to provide them with relevant information, etc.) At the same time, it looks like much less was done to guarantee the comfort of teachers in e-learning or to make them feel confident with the digital competencies required to work online. 

Universities did provide their teachers with instruction on Google tools or Moodle, but all additional e-learning platforms and distance learning methods, which were supposed to facilitate online education, were explored by the teachers themselves. For some of them (especially those less experienced in working with computers), it turned out to be a rather difficult challenge. Even though the ordinances issued by the authorities of the analyzed Polish university were rendered to the public rather quickly, due to the dynamic COVID-19 situation, it was still impossible to implement a firm and long-lasting strategy for distance learning. 

The uncertainty of the situation, which changed rapidly and constantly, caused confusion with respect to conducting the classes. Between March 2020 and March 2022, there were short periods when the didactic process was conducted stationarily. However, those periods can be considered exceptions, not standards. Nevertheless, the university authorities, along with the ministry responsible for higher education, aimed at sustaining the teaching process and making the transformation to distance learning as smooth as it could be. 

The distance education experience in many countries worldwide was challenging, yet similar. Teachers needed to learn how to conduct classes effectively with or without the support of the state or any organizations. The worked-out methods, however, may be valuable for the future of distance learning.

This study has two significant practical implications. First, it can be used for further, more extensive research on implementing hybrid learning in crisis situations and comparing how it was conducted in different European countries. Second, findings from this study can serve (i) as a basis for further research dedicated to the analysis of the effects of hybrid learning implementation on student satisfaction and (ii) as a foundation for the development of a framework for introducing distance learning and then hybrid learning in crisis situations.

## 5. Conclusions

This study aimed at analyzing directions given to higher education teachers regarding the shift to distance learning in Poland. The study was based on a content analysis of acts and regulations issued by the government, university consortia, and universities individually. Based on the content analysis, the findings show that shifting to distance learning was a long-lasting process, which was adjusted multiple times during a two-year period. In our study, we have identified a six-step process run at the University of Economics in Katowice to adjust to distance teaching. These steps are: online platforms for education (1), live contact (2), online semester exams (3), online bachelor’s and master’s exams (4), quality improvement (5), and hybrid education (6). The findings of the study can be used for further and more comprehensive research into the implementation of distance learning in crisis situations, and they may serve as a basis for developing a framework to introduce distance learning followed by hybrid learning in crisis situations. This study has provided a foundation for further research dedicated to the analysis of the effects of hybrid learning implementation.

The most important limitation that this study met was the example of actions presented only by one university. However, this approach was presented in detail. An interesting approach would undoubtedly be to analyze a group of universities, for example, from one geographical region or one type of university throughout the country. This would provide less general and more diversified results because it would create specific questions with respect to the region or university type. The homogeneity of economics universities limits this study’s application to the general public; therefore, it only contributes to higher education.

## Figures and Tables

**Table 1:** Number of messages with the authors and type of communication.

Author	Type of Communication	All	COVID Related
Ministry of Education and Science	Legal act	187	57
Ministry of Education and Science	Website news	1145	565
National Conference of Rectors (KRASP)	Ordinances	3	3
University of Economics in Katowice	Website news	468	140
University of Economics in Katowice	Newsletter	56	56
University of Economics in Katowice	Rector’s ordinances	353	15
University of Economics in Katowice	Rector’s announcements	15	15

**Table 2:** Time-lined summary of the distance learning government regulations introduced in Poland at the beginning of the COVID-19 pandemic.

Date	Summary of Government Regulations
11 March 2020	Pandemic in Poland was announced
20 March 2020	Teaching activities at universities throughout the country were suspended
23 March 2020	Suspension was prolonged; distance learning form suggested
26 March 2020	Online exam conduction was approved by the Ministry
27 March 2020	Official recommendations on distance learning were issued by the Ministry
1 April 2020	Free online course about distance learning methods for educators started
15 September 2020	Hybrid model was recommended as a method of education organization

**Table 3:** Time-lined steps of distance learning introduced at the University of Economics in Katowice, Poland, according to governmental and institutional regulations.

Date	Action Taken by UE Katowice
STEP 1—online platforms for education	
11 March 2020	Suspension of classes and introduction of distance learning
	Establishment of the Crisis Management Committee
13 March 2020	Obligatory use of Google Classroom or Moodle
	Unification of the email account for students and employees in “Google for schools”
21 March 2020	Summary of the rules for the distance learning was issued
	Instructional videos on the use of Google services were published
STEP 2—live contact	
30 March 2020	UE strategy of transition to distance learning was announced
	ICT for teacher–student live contact were introduced
STEP 3—online semester exams	
3 April 2020	Detailed rules for conducting semester exams with the use of ICT were issued
	Opinion poll on e-learning for students was conducted
STEP 4—online bachelor’s and master’s exams	
17 April 2020	Decision to conduct online bachelor’s and master’s exams
21 March 2020	Instructional videos on the use of Google services were published
STEP 5—quality improvement	
18 September 2020	G Suite Platform is obligatory regardless of the form of study
	Requirement for interactive contact with the student using Google Meet
	Hybrid teaching for studies conducted in English was introduced
	International exchange and doctoral studies—stationary form announced
	Teachers were obliged to invite a coordinator to verify the quality of classes
15 October 2020	Stationary activities were suspended
January 2021	Polish Accreditation Commission—accreditation process online
8 February 2021	Announcement that full-time teaching can be restored, or hybrid teaching introduced
STEP 6—hybrid education	
29 September 2021	Introduction of hybrid education in winter semester 2021/2022
2 February 2022	Hybrid education in the summer semester 2021/2022

## Data Availability

Not applicable.
